# Close Relationship between cIAP2 and Human ARDS Induced by Severe H7N9 Infection

**DOI:** 10.1155/2019/2121357

**Published:** 2019-04-07

**Authors:** Chu Qin, Xiao-yan Sai, Xiu-fen Qian, Yan Wu, Li-fei Zou, Hong-mei Wang, Tao Bian, Zheng Yan

**Affiliations:** ^1^Department of Respiratory Medicine, Wuxi People's Hospital Affiliated to Nanjing Medical University, 299 Qingyang Road, Wuxi 214000, Jiangsu, China; ^2^Department of Critical Care, Wuxi People's Hospital Affiliated to Nanjing Medical University, 299 Qingyang Road, Wuxi 214000, Jiangsu, China

## Abstract

**Background:**

cIAP2 is involved in necroptosis as a key upstream regulation factor. We aimed to investigate the role of cIAP2 in ARDS/ALI induced by H7N9 virus through regulating the RIPK1/3 necroptosis pathway.

**Methods:**

Lung tissues of 11 patients who died from ARDS-complicated H7N9 infection between 2013 and 2016 were obtained as the H7N9-ARDS group. Lung tissues near benign lung nodules were acquired as the control group. Histological changes were evaluated by H&E staining. Protein levels of cIAP2, RIPK1, RIPK3, p-RIPK3, MLKL, and p-MLKL in the lung tissues were detected by Western Blot. The mRNA levels of cIAP2, RIPK1, and RIPK3 were detected by real-time PCR.

**Results:**

H7N9 virus infection had a high mortality, with ARDS being the leading cause of death. The protein level of cIAP2 in the experimental group was lower than that in the control group (P<0.05). However, the experimental group showed higher RIPK1, RIPK3, and p-RIPK3 protein levels than the control group (P<0.05), as well as the expression level of MLKL and p-MLKL protein, which is a key downstream protein in necroptosis (P<0.05).

**Conclusion:**

In tissues from patients with fatal H7N9, downregulation of cIAP2 and induction of necroptosis was observed. We could speculate that necroptosis of the pulmonary epithelium is associated with severe H7N9 infection leading to ARDS. Thus, necroptosis inhibition may be a novel therapy for H7N9 influenza virus.

## 1. Introduction

Human infection with novel influenza A H7N9 virus was first reported in March 2013 [1]. Over 600 new cases of infection have been reported for the last few years [2]. The extremely high mortality of human infection with A/H7N9 virus, which was over 40%, seriously endangered the health of human beings and raised great panic [3]. There was no effective treatment for this acute respiratory infectious disease. Thus, it is of great importance now to investigate the pathophysiology of A/H7N9 virus infection, especially that in severe cases, and develop new treatment strategies.

This emerging A/H7N9 virus mainly targets the lungs of humans and causes a rapidly progressive respiratory infection [4]. The incidence of acute lung injury (ALI)/acute respiratory distress syndrome (ARDS) was much higher in H7N9 infection cases than in ordinary flu. ALI/ARDS is the most common cause of death in human infection with A/H7N9 virus [5, 6]. Apart from cytokine storm, cell death of pulmonary epithelia and infected cells played an important role during the course of ALI/ARDS [7–9]. Necroptosis, also named as programmed necrosis, is identified as a new form of cell death. Recent studies have shown that necroptosis was closely connected with a wide range of diseases, including tumor and brain injury [10, 11]. However, whether necroptosis participated in ALI/ARDS induced by A/H7N9 virus in human remains unclear.

As critical upstream regulatory factors in necroptosis pathway, cellular inhibitors of apoptosis proteins (cIAPs) play a significant role in inflammation and innate immunity through its E3 ubiquitin ligases [12–14]. cIAP2 is an essential member of IAPs family. Its combination with receptor-interacting protein kinase 1 (RIPK1) could activate NF-*κ*b and MAPK pathways for cell survival [15]. cIAP2 depletion may trigger the necroptosis pathway and lead to the formation of necrosome combined with RIPK1 and receptor-interacting protein kinase 3 (RIPK3) accompanied by the recruitment and activation of mixed lineage kinase domain-like protein (MLKL) [16–18]. Rodrigue et al. found that cIAP2 knocked out mice had higher mortality and was more sensitive to acute lung injury induced by influenza H1N1 virus. cIAP2 could protect lung tissues from being injured by influenza virus infection and promote host survival [19]. Therefore, cIAP2 has close relationship with acute lung injury induced by influenza virus.

Nowadays, little is known about the regulatory mechanism of ALI/ARDS in severe human infection with A/H7N9 virus. In this study, we found that downregulation of cIAP2 and necroptosis of the pulmonary epithelium is associated with severe H7N9 infection leading to ARDS.

## 2. Methods

### 2.1. Ethics Statement

This study was approved by the Ethics Committee of Wuxi People's Hospital affiliated to Nanjing Medical University. Each patient participating in this study has signed a written informed consent by themselves and/or their families. Each experiment was performed 3 times with 3 replicates by one researcher.

### 2.2. Patients and Lung Tissue Sampling

According to the diagnosis and treatment plan for human infection with H7N9 published by National Health and Family Planning Commission of the People's Republic of China, combined with the positive results of A/H7N9 virus nucleic acid tested by Wuxi Center for Disease Control and Prevention, 22 patients were diagnosed as human avian influenza A H7N9 infection since 2013 in Wuxi. Once the infection was confirmed, these patients were hospitalized in Wuxi People's Hospital and included in this study.

11 patients were complicated with acute respiratory distress syndrome (ARDS) and ended up dead. After they were declared death, their lung tissues were immediately obtained through a disposable lung biopsy needle from the right lung. In addition, according to the match of age and sex, another 10 patients' normal lung tissues were acquired as a control group during their resection surgery of benign lung nodules. Once obtained, a small portion of the lung tissue was fixed in 10% formaldehyde for histological evaluation, the rest were stored in -80°C freezer for further analysis.

### 2.3. Histopathology and Lung Injury Evaluation

The lung tissues fixed in 10% formaldehyde were then embedded in paraffin and cut in thin sections (4-*μ*m). The sections were stained with hematoxylin and eosin (HE) by a standard protocol. Lung injury scores were evaluated by professional pathologists according to the following features: (1) hyaline membrane formation and/or broadened alveolar septum, (2) alveolar congestion, (3) loss and disordered arrangement of bronchial epithelium cells, and (4) infiltration of inflammatory cells in vascular wall and/or alveolar space. Each pathological feature was scored by a 5-scale scale: 0=no injury or very mild injury, 1=mild injury, 2= moderate injury, 3=severe injury, and 4=very severe injury. The total lung injury score of each section was the sum of each score.

### 2.4. Cell Culture and Treatment with cIAP2 Inhibitors

MLE-12 (mouse alveolar epithelium cells) was obtained from Chi Scientific. Cells were cultured in H-DMEM (Gibco, USA) with 10%FBS (Gibco, USA) and antibiotics (100 units/ml penicillin and 100 mg/ml streptomycin) in a humidified atmosphere with 5% CO2/95% air at 37°C. MLE-12 cells were treated with LPS (Sigma Aldrich, USA) (25 *μ*g/ml) for 24h in culture medium. Cells were treated with SM-164 (cIAP2 inhibitor (MCE, USA) at 10uM for 24h in the absence and presence of LPS.

### 2.5. Cell Viability Assay

To perform the MTT assay 3,000 to 3,500 cells per well were plated in 96-well sterile plastic plates and allowed to attach overnight; the cells were then exposed to SM-164 for 24h with or without LPS for 24h, and 20 *μ*l thiazolyl blue (MTT, Sigma, USA) was added in each well. After incubation for 4 h at 37°C, the supernatant was removed and 150 *μ*l DMSO (Corning, USA) was placed in each well to dissolve formazan for 10 min with gentle shaking at room temperature. The absorbance of each sample was measured at 490 nm. The average of three repeated experiments was calculated.

### 2.6. RNA Preparation and Real-Time PCR Analysis

Total RNA of lung samples was extracted using the RNAiso Plus Reagent (Takara, Japan). Reverse transcription of RNA into cDNA was performed through a PrimeScript RT reagent kit with gDNA Eraser (Takara, Japan). The mRNA expression level in lung tissues was than quantified by real-time PCR analysis using a SYBR Premix Ex TaqTMII (Takara, Japan). The primers used were as follows: cIAP2(F): GGGACCAACAGGTTGTTCTGGTA, cIAP2(R): CAGAGTTATGACTCGGACGTGTTGA, RIPK1(F): GGGAAGGTGTCTCTGTGTTTC, RIPK1(R): CCTCGTTGTGCTCAATGCAG, RIPK3(F): CATAGGAAGTGGGGCTACGAT, RIPK3(R): AATTCGTTATCCAGACTTGCCAT, *β*-actin(F): TGGCACCCAGCACAATGAA, *β*-actin(R): CTAAGTCATAGTCCGCCTAGAAGCA.

### 2.7. Western Blot

A suitable amount of lung tissue stored in -80°C freezer was homogenated and lysed in RIPA buffer containing proteinase and phosphatase inhibitor cocktail (CWBIO Co. Ltd., China). After protein concentrations were assessed using the BCA protein assay kit (CWBIO Co. Ltd., China), protein samples were boiled in loading buffer for 10 min. 40 *μ*g protein samples were then run on a 10% SDS-PAGE and transferred onto a PVDF membrane (Millipore, USA) at 250mA for 2h. The membrane was blocked with 5% fat-free milk in TBST (0.1% Tween-20 in TBS) for 2h at room temperature and incubated with primary antibodies at 4°C over night. Primary antibodies used in this study were anti-cIAP2 antibody(1:1000, R&D system), anti-RIPK1 antibody (1:1000, Novus), anti-RIPK3 antibody (1:1000, Abcam), anti-MLKL (1:1000, Abcam), anti-RIP3 (phospho S227) antibody (1:2000, Abcam), anti-MLKL (phospho S345) antibody (1:1000, Abcam), and anti-GAPDH antibody(1:4000, CWBIO Co. Ltd). Then the membranes were washed 3 times in TBST and incubated with goat-anti-mouse/rabbit antibody for 2h at room temperature. After being washed 3 times in TBST again, the protein bands were visualized by fluorography using ECL (enhanced chemiluminescence) reagents (Millipore, USA) and analyzed by image J.

### 2.8. Statistical Analysis

Statistical analysis was performed using SPSS 23.0 and GraphPad Prism 6.01. Quantitative data was expressed as mean±SEM or SD. Analysis of difference between two groups was carried out with Independent-Samples T test. Count data was expressed as constituent ratio and compared by chi-test or Fisher exact probability method. P< 0.05 was considered to be statistically significant.

## 3. Results

### 3.1. Human Infection with H7N9 Virus Had a Poor Prognosis

Between April 2013 and January 2017, a total of 22 patients were diagnosed as human infection with H7N9 virus and hospitalized in Wuxi People's Hospital (Table 1). Every patient started with a fever. Notably, 50% of these patients (11/22) ended up dead, which revealed a fairly high mortality of this disease. The mortality between different ages and sex had no difference (P>0.05). Besides, 27.27% of the patients (6/22) had clear history of poultry exposure, which might have no relevance with the high mortality (P>0.05). Moreover, 45.45% of the patients (10/22) had underlying diseases, such as hypertension, diabetes, chronic bronchitis, and so on. However, there was no difference in the patients' mortality with or without underlying diseases (P>0.05).

### 3.2. ARDS Was a Leading Cause of the High Mortality of Human Infection with H7N9 Virus

In this study, the incidence of ARDS in human infection with avian influenza A H7N9 virus was 68.18% (15/22). The mortality of patients complicated with ARDS was 73.33% (11/15), which was significantly higher than patients without ARDS (P<0.05). Among these patients with ARDS, different ages and sex, as well as underlying diseases and clear history of poultry exposure, had no significant effect on the high mortality (P>0.05).

### 3.3. Pathological Changes of the Lung Tissues

**Figure 1:**
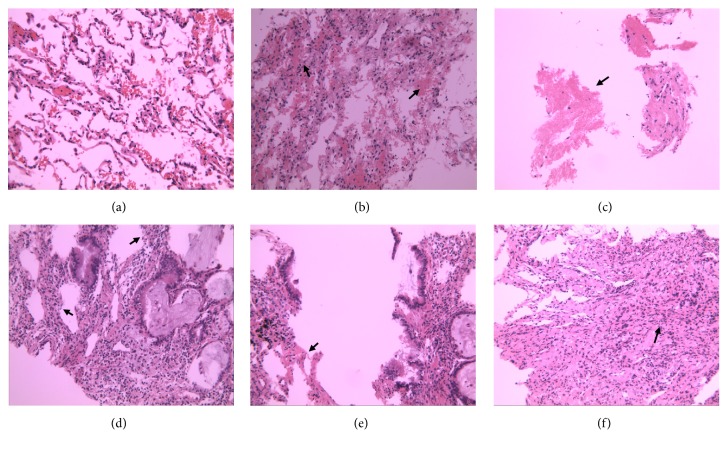
Pathological changes in the lung tissues (H&E staining, 200x). The lung tissues in control group had few abnormal pathological changes (a). The experimental group showed severe alveolar congestion (b), exudation of fibrin (c), broadened alveolar septum (d), disordered arrangement of bronchial epithelium (e), and inflammatory cells infiltration (f).

**Figure 2:**
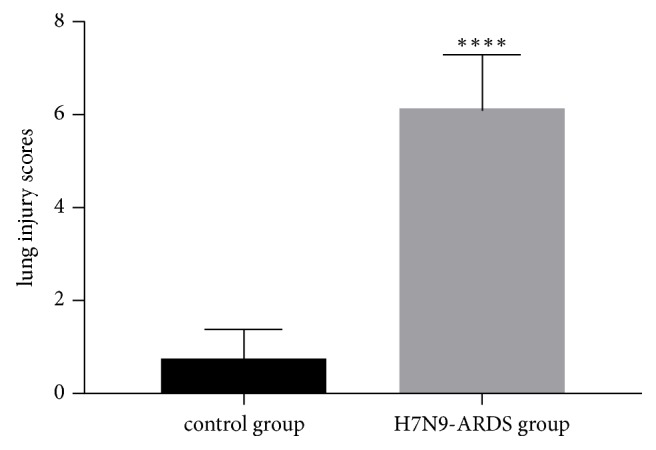
Lung injury scores. Each pathological feature was scored by a 5-scale point: 0=no injury or very mild injury, 1=mild injury, 2= moderate injury, 3=severe injury, and 4=very severe injury. The total lung injury score of each section was the sum of each score. The lung injury score of the experimental group was significantly higher than the control group (P<0.05).

H&E staining showed that the lung tissues in control group had few abnormal pathological changes without inflammatory cell infiltration and alveolar abnormality. However, the lung tissues of the patients killed byavian influenza A H7N9 virus exhibited severe alveolar congestion, marked infiltration with inflammatory cells, and broadened alveolar septum. We also observed disordered or loss of arrangement of bronchial epithelium in the experimental group (Figure 1). The lung injury score of the experimental group was 6.1 ± 0.38, which was significantly higher than the control group (0.7 ± 0.21) (P<0.05, Figure 2).

### 3.4. cIAP2 Expression Was Inhibited in the Lung Tissues of Patients Dying from Human Infection with H7N9 Virus

**Figure 3:**
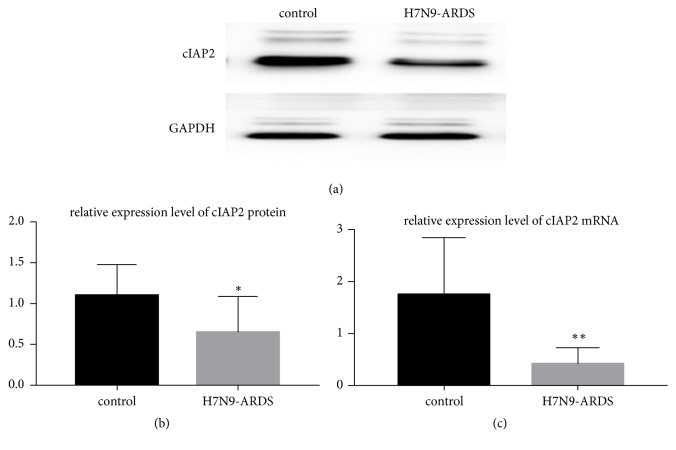
cIAP2 expression level. cIAP2 protein had a lower expression level, in the lung tissues of the patients dying from human infection with H7N9 virus than the control group. ((a) and (b) P<0.05). The experimental group exhibited a lower cIAP2 mRNA level than the control group. ((c) P<0.05).

We measured cIAP2 mRNA and protein level to assess the cIAP2 expression pattern in the lung tissues. It turned out that the experimental group exhibited a lower cIAP2 mRNA level than the control group (P<0.05, Figure 3(c)). Similarly, cIAP2 protein had a notably lower expression level, sometimes no expression, in the lung tissues of the patients dying from human infection with H7N9 virus than the control group (P<0.05, Figures 3(a) and 3(b)).

### 3.5. RIPK1/3 Mediated Necroptosis Was Found in the Lung Tissues of Severe Patients Dying from Human Infection with H7N9 Virus

**Figure 4:**
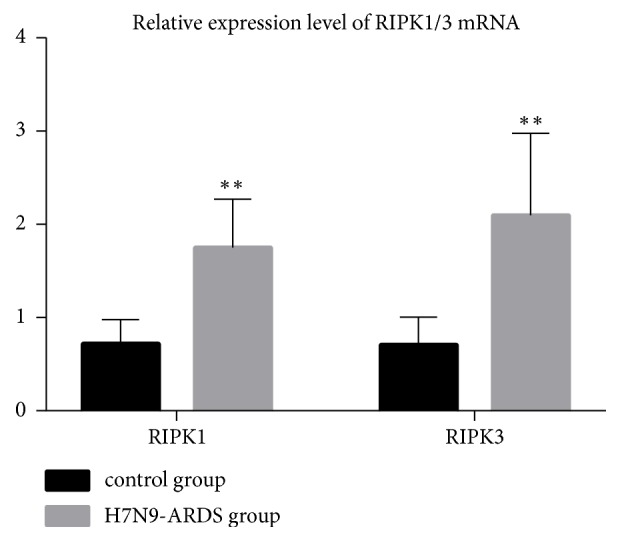
The mRNA level of RIPK1 and RIPK3. The mRNA level of RIPK1 and RIPK3 was enhanced in the lung tissues of severe patients dying from human infection with H7N9 virus (P<0.05).

**Figure 5:**
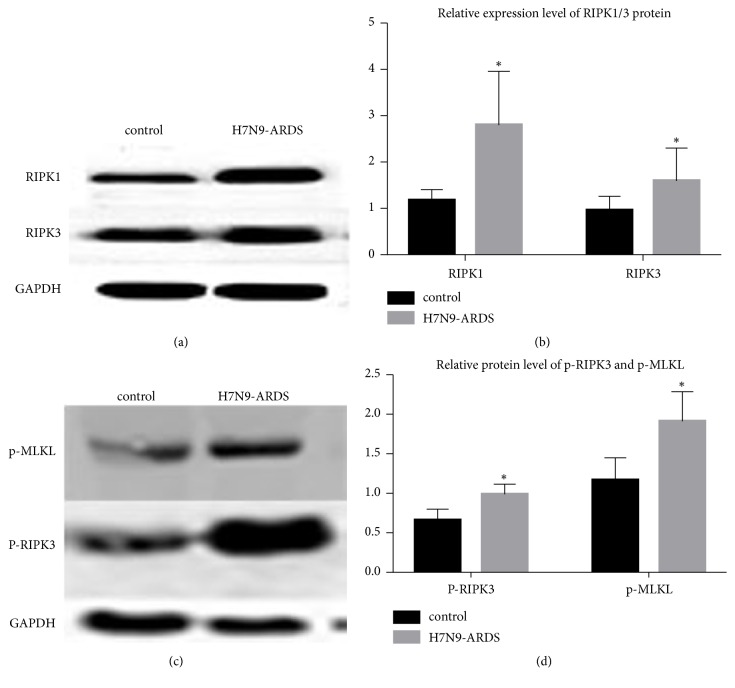
The expression of RIPK1 and RIPK3. The protein level of RIPK1 and RIPK3 was increased in the lung tissues of severe patients dying from human infection with H7N9 virus (P<0.05). The protein level of p-RIPK3 and p-MLKL was increased in the lung tissues of severe patients dying from human infection with H7N9 virus (P<0.05).

The necroptosis pathway was activated by the formation of necrosome, which was up to the combination of RIPK1 and RIPK3. In this study, the mRNA level of RIPK1 and RIPK3 was enhanced in the lung tissues of patients killed by infection with H7N9 virus (P<0.05, Figure 4), as well as the protein level of RIPK1 and RIPK3 (P<0.05, Figure 5). We also found an elevated level of RIPK3 serine 227 phosphorylation in the lung tissues of the H7N9-ARDSl group than that in the control group (P<0.05, Figure 5).

**Figure 6:**
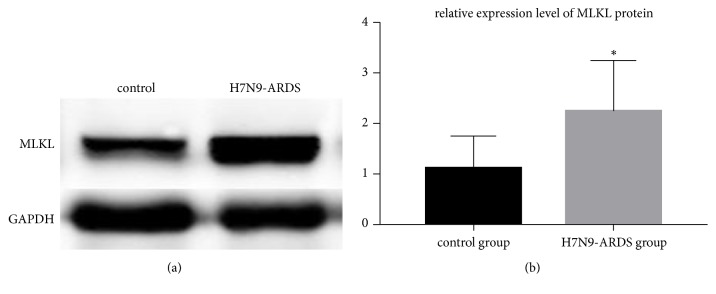
The protein level of MLKL. The protein level of MLKL was increased in the lung tissues of severe patients dying from human infection with H7N9 virus (P<0.05).

Moreover, we observed increased expression of MLKL protein, which is a key downstream marker for necroptosis (P<0.05, Figure 6). Meanwhile, phosphorylation of MLKL (S345) was also enhanced in the lung tissues of patients killed by infection with H7N9 virus (P<0.05, Figure 5).

### 3.6. Necroptosis Was Observed in SM-164 Treated Alveolar Epithelium Cells during LPS-Induced ARDS

**Figure 7:**
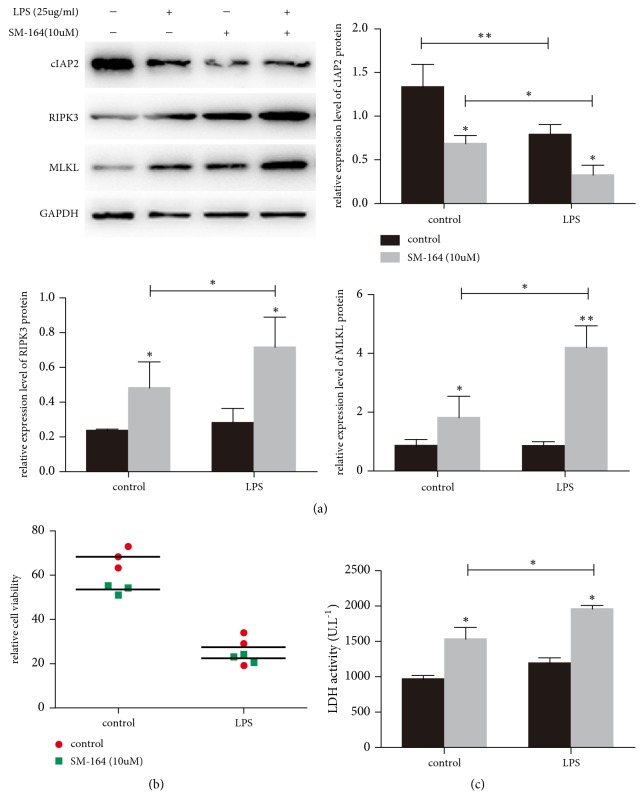
Necroptosis was observed in SM-164 treated alveolar epithelium cells during LPS-induced ARDS. The mouse alveolar epithelium cell line MLE12 was exposed to SM-164 for 24h with or without LPS for 24h. RIPK3 and MLKL were downregulated along with the inhibition of cIAP2 by SM-164 during LPS-induced ARDS (a). The loss of viability is correlated with cell death as detected by LDH release in the cell supernatant ((b) and (c)). ^*∗*^p<0.05.

The mouse alveolar epithelium cell line MLE12 was treated with SM-164 for 24 h. SM treatment degraded cIAP2 at the concentration of 10uM. We also use LPS treatment (25ug/ml) to induce ARDS model. We utilized the MTT assay to confirm loss of cell viability with SM for 24 h (Figure 7(b)). Besides, the loss of viability is correlated with cell death as detected by LDH release in the cell supernatant (Figure 7(c)). We found that RIPK3 and MLKL was downregulated along with the inhibition of cIAP2 by SM-164 during LPS-induced ARDS (Figure 7(a)). Thus, it appears that cIAP2 inhibition could activate necroptosis by the formation of necrosome.

## 4. Discussion

The emerging H7N9 virus is a subtype of influenza virus and recombined by classical H7N9, H7N3, and H9N2 [1, 9, 20]. Recently, there were several outbreaks of human infection with H7N9 virus accompanied with over 40% mortality around the world, especially in China. Since 2013, 22 cases of human infection with H7N9 virus have been admitted into Wuxi People's Hospital. 11 cases among them ended up dead. The high mortality greatly raised our interest in further exploration of the cell death pattern and finding new treatment for this threatening respiratory virus infectious disease.

Compared with common influenza, human infection with H7N9 virus had a significantly higher incidence of ALI, which may rapidly develop into ARDS and multiple organ dysfunction. ALI/ARDS was the leading cause of the death of severe human infection with H7N9 virus [21, 22]. In this study, we found that it was quite often for patients infected by H7N9 virus to be complicated with ARDS. The mortality of patients complicated with ARDS was significantly higher than those without ARDS. Meanwhile, ARDSoccurred in each dead patient infected with H7N9 virus. Histopathological study showed that severe alveolar congestion, marked infiltration with inflammatory cells, broadened alveolar septum, and disordered arrangement of bronchial epithelium cells were observed in the experimental group. Huang et al. reported pneumocyte hyperplasia and diffuse alveolar damage in a critically ill patient with avian influenza A (H7N9). They also observed progressive and rapid lung fibrosis in the lung tissues of that patient who finally died [23]. Likewise, another study reported acute diffuse alveolar damage and pulmonary fibroproliferative changes in lung tissues of fatal human infections with H7N9 virus [24]. These results, which coincided with our findings, revealed the typical pathological changes of ARDS induced by human infection with H7N9 virus.

Cell death of pulmonary epithelia is a critical part in the development and progress of ALI/ARDS [7]. Necroptosis, a newly discovered form of cell death first reported and named by Degterev et al., was reported to have essential roles in various diseases [25]. Several studies have shown the close relationship between necroptosis and ALI/ARDS [26, 27]. Pan et al. implicated that necroptosis was activated in the oleic acid -induced ARDS rat model [28]. Wang et al. found that lung tissue necroptosis significantly increased in high dose LPS-induced ARDS mouse model [26], whereas there were few researches on the role of necroptosis in ALI/ARDS induced by H7N9 virus in human.

cIAP2 plays an important role on the regulation of necroptosis mainly through its ability to promote cell survival [29–31]. Rodrigue et al. reported that cIAP2 could protect mice from influenza infection by antagonizing RIPK1/3-mediated necroptosis to promote host survival [19]. In this study, we found that cIAP2 was significantly downregulated in the lung tissues of patients killed by infection with H7N9 virus. In contrast, cIAP2 was upregulated in ALI mice model induced by low dose LPS (5mg/kg) (data not shown). This implied that apoptosis instead of necroptosis was remarkably increased in low dose LPS-induced mild ARDS. This result was in accordance with the study carried out by Wang et al. [26]. These results indicated that the necroptosis pathway regulated by cIAP2 may be involved in ALI/ARDS in severe human infection with H7N9 virus cases.

During the process of necroptosis, the combination of cIAP2 and RIPK1 is inhibited and switched to the formation of necrosome, which is combined by RIPK1, RIPK3, and TRADD. RIPK1 and RIPK3 phosphorylate and combine with each other. MLKL is then phosphorylated and recruited as a substrate to trigger RIPK1/3-mediated necroptosis [32, 33]. Recently, several studies have reported that expression levels of RIPK1, RIPK3, and MLKL were increased, which confirmed the presence of necroptosis and finally led to cell death [26, 34]. Additionally, phosphorylation of RIPK3 and MLKL has also been recognized as the hallmark of necroptosis [34]. In the present study, we showed that RIPK1 and RIPK3 were upregulated in lung tissues of severe patients dying from human infection with H7N9 virus. Besides, phosphorylated RIP3 (p-RIP3) was increased in the experimental group compared to that in the control group. Moreover, we observed a raised protein level of both MLKL and p-MLKL in lung tissues of severe patients dying from human infection with H7N9 virus. These results revealed that necroptosis may exist in the lung tissues of ARDS patients induced by H7N9 virus. Unlike our results, another research by Xu et al. demonstrated that caspase 1/IL-1*β* signaling was involved in RIP3-associated inflammation of influenza H7N9 virus instead of RIP3/MLKL necroptosis. They found that RIPK3 was increased in mice exposed to H7N9 virus. However, there was no significant difference of MLKL expression level between H7N9-infected WT and RIP3-/- mice [35]. Interestingly, Mohsen et al. observed that necroptosis-related molecules had significant different expression between human and mice [36]. The species-specific expression of necroptosis-related molecules could explain the contradiction between our study and the research by Xu et al. [35].

## 5. Conclusions

This study indicated that human infection with H7N9 virus had an extremely high mortality. ALI/ARDS was the leading cause of death in severe cases. The occurrence of ALI/ARDS in each severe case may be resulted from the low expression of cIAP2, which could lead to the increased combination of necrosome formed by RIPK1 interacting with RIPK3. MLKL, the downstream substrate, was then recruited and activated. We suspect that the abnormal expression of cIAP2 caused the activation of RIPK1/3-dependent necroptosis, which triggered death of airway epithelial cells and resulted in ALI/ARDS and death. The specific mechanism needs further investigation.

## Figures and Tables

**Table 1:** Clinical features of 22 cases of human infection with H7N9 virus.

No.	Sex	Age	poultry exposure history	Fever	dyspnea	cough	Underlying diseases	ARDS	Prognosis
(1)	male	61	Not clear	+	+	+	hypertension	+	dead
(2)	female	33	Not clear	+	-	+	-	+	dead
(3)	female	63	Not clear	+	+	-	-	+	dead
(4)	female	60	Not clear	+	+	+	aplastic anemia	+	dead
(5)	female	36	Not clear	+	-	+	-	+	dead
(6)	male	35	Not clear	+	-	+	-	+	dead
(7)	female	27	+	+	+	-	-	+	dead
(8)	male	70	+	+	+	-	hypertension	+	dead
(9)	female	63	+	+	-	+		+	dead
(10)	male	76	Not clear	+	+	+	hypertension	+	dead
(11)	male	77	Not clear	+	+	+	hypertension, Type 2 diabetes	+	dead

(12)	male	74	Not clear	+	-	+	AECOPD,hypertension, Corpulmonale	+	alive
(13)	male	66	Not clear	+	+	+	hypertension, chronic bronchitis	-	alive
(14)	female	47	+	+	+	+	-	+	alive
(15)	female	49	Not clear	+	+	+	-	+	alive
(16)	male	46	Not clear	+	-	+	-	+	alive
(17)	male	61	+	+	-	+	hypertension	-	alive
(18)	male	66	-	+	-	+	Type 2 diabetes	-	alive
(19)	male	48	+	+	+	+	-	-	alive
(20)	female	59	-	+	+	+	-	-	alive
(21)	male	40	Not clear	+	+	+	hypertension	-	alive
(22)	male	43	-	+	-	+	-	-	alive

## Data Availability

The reported data used to support the finding of this study are available from the corresponding author upon request.
